# Robust strong coupling of single quantum emitters with plasmonic nanocavity on one-dimensional photonic crystal substrate

**DOI:** 10.1016/j.fmre.2025.04.012

**Published:** 2025-04-29

**Authors:** Bowen Fu, Longlong Yang, Yu Yuan, Jingnan Yang, Hancong Li, Zetao Fan, Sai Yan, Guowei Lu, Douguo Zhang, Qihuang Gong, Xiulai Xu

**Affiliations:** a State Key Laboratory for Mesoscopic Physics and Frontiers Science Center for Nano-optoelectronics, Peking University, Beijing 100871, China; b Beijing National Laboratory for Condensed Matter Physics, Institute of Physics, Chinese Academy of Sciences, Beijing 100190, China; c Advanced Laser Technology Laboratory of Anhui Province, Department of Optics and Optical Engineering, University of Science and Technology of China, Hefei 230026, China; d Peking University Yangtze Delta Institute of Optoelectronics, Nantong 226010, China; e Collaborative Innovation Center of Extreme Optics, Shanxi University, Taiyuan 030006, China

**Keywords:** Plasmonic nanocavity, Single quantum dot, Strong light-matter interaction, Photonic crystal cavity, Rabi splitting

## Abstract

The plasmonic nanocavity with extremely localized field distribution can facilitate strong coupling with emitters at the level of single quanta. However, the dramatic variation of the plasmon field with distance on the surface of nanocavity makes the strength of the interaction very sensitive to the location of the individual emitter. Here, we present a new strategy that achieves room-temperature strong coupling by integrating a single CdSe/ZnS quantum dot with a bowtie-structured plasmonic nanocavity on a one-dimensional photonic crystal (1DPC) substrate. This kind of hybrid cavity enables a more uniform field distribution in the nanogap, which is more robust and stable. The Rabi splitting with single exciton reached ∼170 meV and the lifetime of photon emission is reduced by ∼5 times. This work provides an new way to construct solid-state cavity quantum electrodynamic system in strong coupling region at room temperature.

## Graphical abstract

**Figure:**
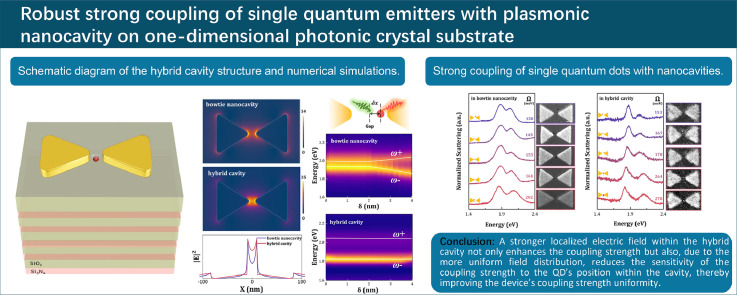

## 1 Introduction

Strong coupling between single quantum emitter and an optical cavity is one of the important research area in cavity quantum electrodynamics (cQED) [[1], [2], [3], [4]], where the strong coupling strength is very crucial for developing various quantum devices, such as the single-photon logic gate [[5], [6]], single-photon transistor [[7], [8]], and the single-photon switch [[9], [10]]. The strong coupling regime is characterized by Rabi splitting, with coupling strength (*g*) quantified by the Rabi splitting $\Omega_{R}$ (where $\Omega_{R}=2g$) [[11], [12]]. Dielectric optical microcavities with high quality factors have been intensively studied for the cQED [[13], [14], [15]]. However, mode volumes are difficult to compress to the same nanoscale as that of the single quantum emitter, which limits the further increase of coupling strength and the applications in ultra-compact integrated quantum optical devices. Plasmonic nanocavities can overcome the diffraction limit by localizing strong electromagnetic fields within a mode volume much smaller than the wavelength of light, which allows the realization of strong light-matter interactions more easily [[16], [17], [18], [19], [20], [21], [22], [23]]. Especially, the bowtie nanocavity with ultra-small mode volume is commonly used for the strong coupling system, in which the field distribution can be easily tuned by changing the gap distance [[24], [25]]. Therefore, integrating such nanocavities with single quantum emitters having a large oscillator strength enables the operation of quantum devices at room temperature [[26], [27]].

Although the extremely concentrated plasmon field enhances the coupling strength and reduces the number of emitters involved in the coupling, the interaction strength is highly sensitive to the emitter’s location, as the electric field decays almost exponentially in the direction perpendicular to the structure’s surface [28]. Coupling with excitons in two-dimensional materials avoids this problem to some extent, because excitons are evenly distributed within atomically thin layered materials, so the excitons can interact with the maximum of the local field directly. However, the number of involved excitons is still in the stage of low exciton number [[22], [29]]. Coupling with dye molecules remains challenging because of the uncertainty in the orientation of the dipole moment [[30], [31], [32], [33]]. The coupling of a single quantum dot (SQD) with optical nanocavities is an effective approach to address this issue, but the positioning of SQD in the nanocavity is still challenging. Despite various strategies reported to integrate a SQD into plasmonic nanostructures [[26], [27], [34], [35]], the non-uniform distribution of the electric field strength within plasmonic nanocavities results in coupling strength being position-dependent, thus affecting the consistency of coupling strength and limiting the further application of such strongly coupled system.

In this work, we fabricate a hybrid cavity by combining a plasmonic nanocavity with a 1DPC to modulate the electric field within the nanocavity, achieving a more uniform electric field distribution. Through dark-field scattering spectroscopy, we demonstrated an enhancement in coupling strength and improved the consistency of the hybrid cavity. With the surface morphology of scanning electron microscopy (SEM) images, we determine the number of quantum dots inside the nanovavities and demonstrate the strong coupling involved with only one SQD. For the deterministic interaction of a single gold bowtie nanostructure and a single quantum dot, a maximum Rabi splitting of approximately 170 meV is obtained. This robust strong coupling system involving the single exciton transition provides a platform for the study of integrated quantum optics.

## 2 Method

### 2.1 Simulation of strong coupling

To investigate the coupling between a single CdSe/ZnS QD and a plasmonic nanocavity, we simulate their scattering spectra using Finite-Difference Time-Domain (FDTD) method. The CdSe/ZnS QD is as a spherical nanoparticle in the simulation, and its dielectric properties are described using a Lorentz model for the dielectric constant [36]:

$\varepsilon_{QD}\left(\omega \right)=\varepsilon_{\infty }-f\frac{\omega_{QD}^{2}}{\omega^{2}-\omega_{QD}^{2}+i\gamma_{QD}\omega }$ where, the background dielectric constant $\varepsilon_{\infty }=5$, the QD resonance frequency $\omega_{QD}$ = 1.96 eV, the QD radiative linewidth $\gamma_{QD}$ = 0.06 eV, and the oscillator strength *f* = 0.8, all of which are within the range of experimental measurements. The permittivity of gold was taken from Johnson and Christy [37], with a thickness of 35 nm.

### 2.2 Device fabrication

In the experiment, the 1DPC serving as the substrate is composed of 5 pairs of Si${}_{3}$N${}_{4}$ (90 nm thick) and SiO${}_{2}$ (120 nm thick) stacked together, with a top SiO${}_{2}$ layer of 180 nm as the defect layer. These layers are stacked onto the ITO glass substrate through inductively coupled plasma chemical vapor deposition (ICPCVD). A single gold bowtie nanostructure is fabricated using electron beam lithography (EBL) and thermal evaporation on the 1DPC, then a gold bowtie structure with a side length of 100 nm and a gap distance of 20 nm was obtained. To fabricate holes within the gaps of the gold nanostructure, a layer of Poly(methyl methacrylate) (PMMA) 495 K was spin-coated at a speed of 3500 rpm for 45 seconds, then the sample was baked at 180 ^o^C for 60 seconds. EBL was then employed to pattern holes precisely within the bowtie gaps of the nanostructure. Following a previously reported procedure [[26], [27]], the sample was vertically immersed in a CdSe/ZnS QD solution to deposit a single QD into the patterned holes.

### 2.3 Optical characterizations

The morphology analysis of the gold bowtie nanostructure and the verification of the number of quantum dots within the gap were performed using SEM. However, the poor conductivity of the 1DPC surface affects the image sharpness. Nevertheless, by focusing only on the area around the gap, it is still possible to determine the position and number of quantum dots. The dark-field scattering spectra of individual gold nanostructures were recorded using a spectrometer [38]. Polarization-dependent measurements were enabled by incorporating a rotatable polarizer into both the excitation and collection pathways. Photoluminescence (PL) measurements were conducted using a 532 nm pulsed laser operating at a repetition rate of 78 MHz. The laser beam was focused through the microscope objective, creating a 4 μm diameter spot on the sample. Time-resolved spectroscopy is collected using a single-photon detector with time-correlated single-photon counting (TCSPC) technique.

## 3 Results and discussion

### 3.1 Schematic illustration and numerical simulation of the coupled system

**Fig. 1:**
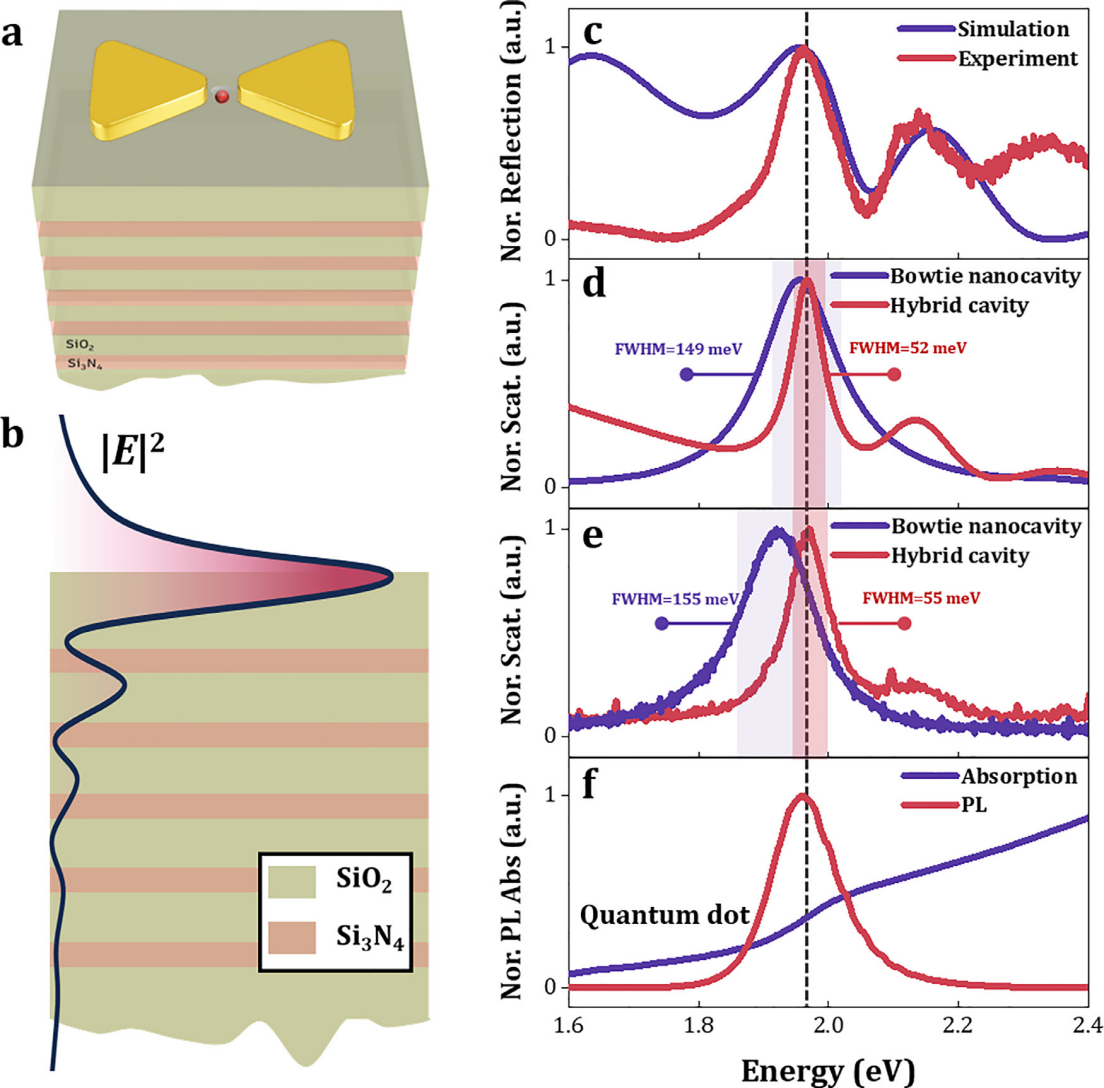
**Design of the hybrid cavity structures.** (a) Schematic of the system: The bowtie nanostructure is positioned on 1DPC substrate, with quantum dots deposited in the gap of the bowtie. (b) Schematic diagram of the dielectric layer stack in the 1DPC and the electric field distribution (black curve), where the electric field distribution is calculated using the continuous transfer matrix method. (c) The reflection spectrum of the 1DPC, with the blue line and red line representing the simulation and experimental results, respectively. (d) and (e) show the plasmonic linewidth compression induced by the 1DPC, with results obtained from simulation and experiment, respectively. (f) The photoluminescence spectrum and absorption spectrum of the quantum dot hexane solution. The dashed lines in (c-f) indicate the energy positions of the quantum dot and the cavity mode. (For interpretation of the references to colour in this figure legend, the reader is referred to the web version of this article.)

A schematic illustration of the system is presented in Fig. 1a. A hybrid cavity comprising a 1DPC cavity and a plasmonic nanocavity was constructed by placing a gold bowtie nanostructure on a 1DPC substrate. A quantum dot was then deposited within the gap of the bowtie nanostructure. The schematic diagram of the multilayer dielectric stack is shown in Fig. 1b. The curve illustrates the electric field distribution within the cavity when the incident light at the wavelength of the QD band, with the strongest electric field distribution occurring at the top of the 1DPC, which is advantageous for coupling with the plasmonic nanocavity on the surface. Multiple intrinsic optical modes are also present in the 1DPC, as observed in the reflection spectrum. The blue and red curves in Fig. 1c represent the simulation and experimental results, respectively, with a cavity mode observed at 1.96 eV, coinciding with the emission position of the quantum dot as shown in Fig. 1f. The electric field pattern of the 1DPC within the hybrid cavity compresses the plasmon mode linewidth (from 149 meV to 52 meV) as shown in Fig. 1d, similar results were also obtained in experiment (Fig. 1e), The linewidth of the hybrid cavity is reduced to 55 meV. The reduced linewidth could be useful to lower the critical conditions for achieving strong coupling by suppressing plasmon damping [[22], [39], [40]].

**Fig. 2:**
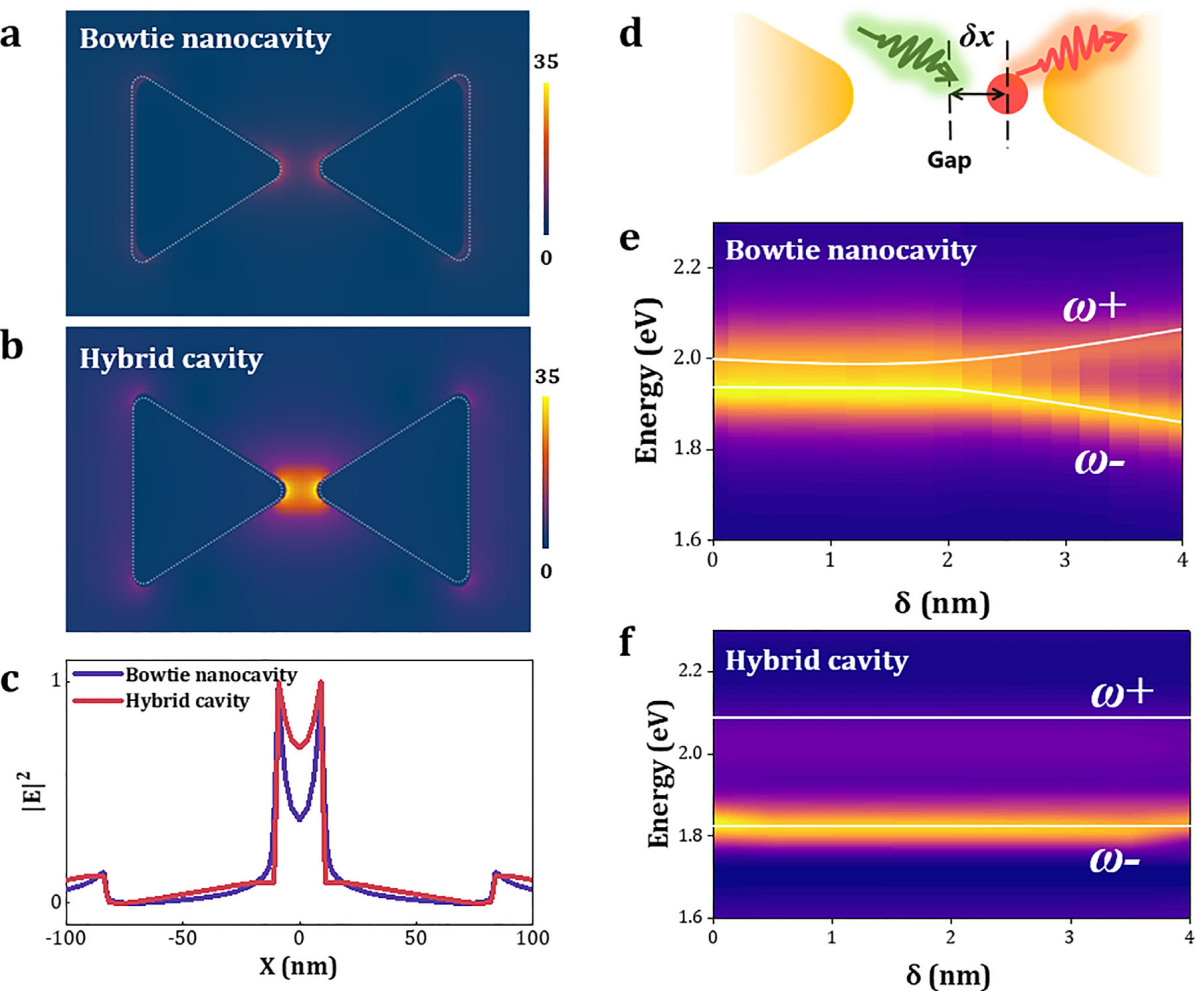
**The electric field distribution and localization in the hybrid cavity.** (a-b) Electric field distribution calculated using FDTD method: (a) bowtie nanocavity and (b) hybrid cavity, with an intensity increase of approximately three times. (c) Normalized electric field distribution along the x-axis, confirming that the hybrid cavity exhibits a more uniform electric field distribution. (d) Schematic diagram of the position of SQD within the gap. Scattering spectra of SQD at (e) bowtie nanocavity and (f) hybrid cavity respectively, with a more stable and larger strong coupling for later case.

To understand the behavior of the coupled system, we used the FDTD method to calculate the electric field distributions within the gold bowtie nanocavity (Fig. 2a) and the hybrid cavity (Fig. 2b) at a resonance energy of 1.97 eV. Here, linearly polarized light at $0^{\circ }$ is used to excite the dipolar mode of the bowtie structure. Unlike the localized electric field near the metal particles in the bowtie nanocavity, the electric field pattern provided by the 1DPC alters the field distribution within the nanocavity: the electric field in the hybrid cavity becomes more uniform across the entire nanocavity, with an intensity enhanced by approximately threefold. The normalized linear electric field distribution along the x-axis in the middle of bowtie is presented in Fig. 2e, corresponding to the same horizontal coordinates as Fig. 2a-b, further confirming the significant improvement in electric field uniformity within the hybrid cavity. This stronger and more uniform field distribution suggests a significant enhancement in both the magnitude and consistency of the coupling strength.

To validate this conclusion, we varied the horizontal position of the single QD within the cavity (Fig. 2d) and simulated the scattering spectra as the QD move further away from the center of the gap (Fig. 2e-f). For the bowtie nanocavity, the Rabi splitting is nearly constant for Δx<2 nm, while it shows a continuous increase for Δx>2 nm due to the closing with gold nanotip. In contrast, the hybrid cavity maintained a nearly consistent splitting magnitude at all positions, exhibiting larger splitting values. These phenomena can be well explained by the electric field intensity and distribution within the nanocavity. It is worth noting that we have only used the positional change of the quantum dots along a single axis as an example for illustration. Based on the results in Fig. 2c, it can be inferred that relatively consistent coupling strength can be maintained over a larger spatial extent within the hybrid nanocavity.

### 3.2 Strong coupling of plasmonic cavity and quantum dots

In the experiment, we employed dark-field scattering spectroscopy to measure the scattering spectra of individual bowtie nanostructures to characterize the plasmonic behavior of each bowtie. To avoid interference from multiple modes within the optical cavity when analyzing the spectral Rabi splitting, a polarizer was used in the experiment to linearly excite the longitudinal mode of the bowtie nanostructure at 1.97 eV, matching the transition energy of the QD. The dark field scattering spectra for several typical bowtie nanostructure devices with QDs embedded within the gap is presented in Fig. 2a and c. All spectra exhibit pronounced Rabi splitting, indicating strong coupling between excitons and photons.

**Fig. 3:**
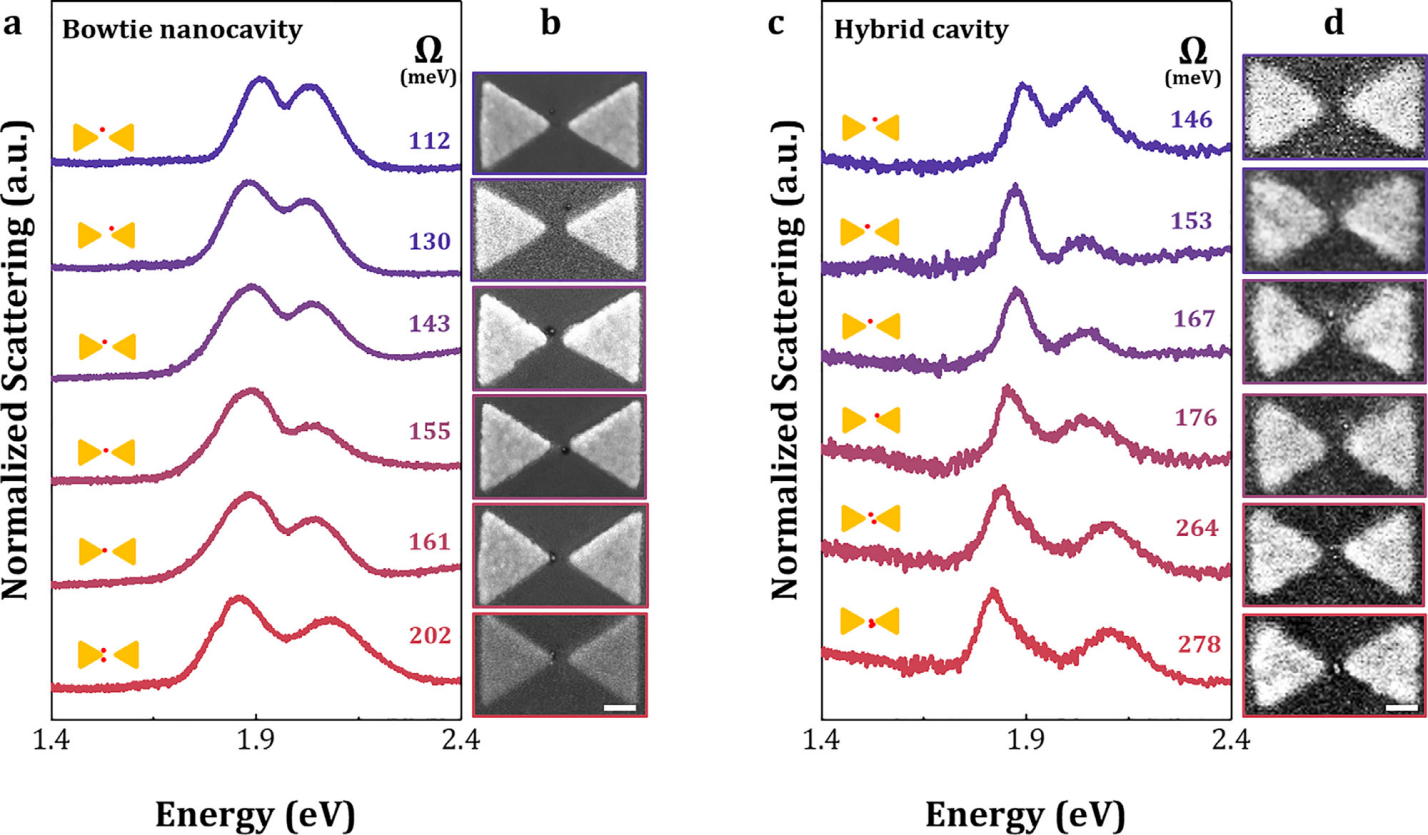
**Strong coupling of single quantum dots with nanocavities.** (a) Dark-field scattering spectra of single quantum dots in the bowtie nanocavity and (b) corresponding SEM images. (c) Dark-field scattering spectra of single quantum dots in hybrid cavity and (d) corresponding SEM images showing single quantum dots located inside the nanocavity. All the SEM images with a scale bar of 40 nm.

In bowtie nanocavities, the Rabi splitting varies significantly among different devices, while the hybrid cavities demonstrate more consistent splitting characteristics. This inconsistency primarily arises from the spatial distribution and quantity differences of QDs within the cavity. Based on SEM images, each device was individually characterized (Fig. 3b and d) to determine the specific positions and quantities of QDs. The results show that in bowtie nanocavities, when the QD located near the tip, the larger electric field intensity in this region leads to enhanced Rabi splitting. In contrast, the splitting decreases in other areas. The maximum and minimum Rabi splitting values measured in the bowtie nanocavities are 161 meV and 112 meV for SQD, respectively, with a difference as large as 49 meV. As for hybrid nanocavities, benefiting from a more uniform and enhanced electric field distribution, exhibit greater consistency in Rabi splitting, with maximum and minimum values of 176 and 146 meV, respectively, and a difference of only 30 meV. Furthermore, a larger Rabi splitting is observed when multiple quantum dots are incorporated within the cavity. For instance, with two quantum dots present in a bowtie nanocavity, the Rabi splitting reaches 202 meV; in the hybrid nanocavity, with two or three quantum dots, the Rabi splitting reaches 264 meV and 278 meV, respectively. This is consistent with the fact that Rabi splitting is proportional to $\sqrt{N}$ (here N represents the number of excitons) in a strongly coupled system [41].

**Fig. 4:**
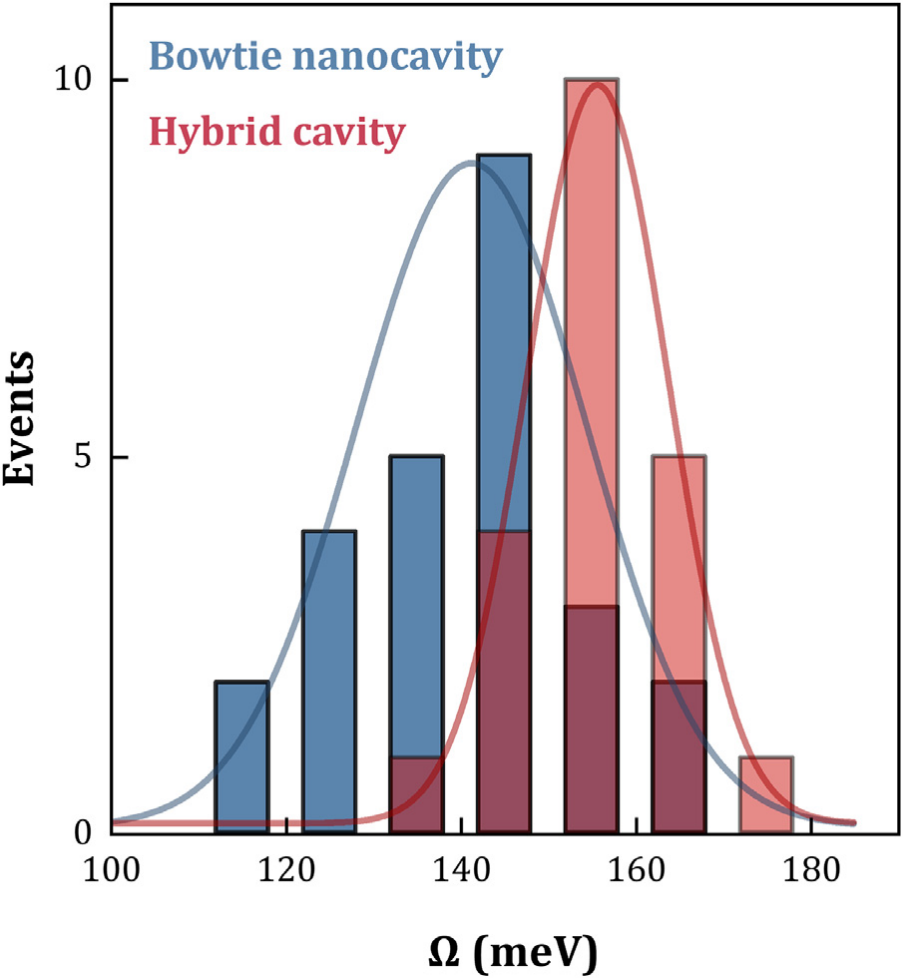
Statistical distribution of Rabi splitting for a single quantum dot in bowtie nanocavities and hybrid cavities, obtained from dark-field scattering spectra.

The statistical distribution histograms of Rabi splitting for 25 bowtie nanocavities (blue) and 21 hybrid nanocavities (red) loaded with a single QD are presented in Fig. 4. The average coupling strength distributions are 140.4 and 156.2 meV, respectively. After fitting the column charts with a Gaussian function, we find that the fitted curves for hybrid nanocavities exhibit narrower full width at half maximum (FWHM), indicating better coupling strength consistency compared to bowtie nanocavities.

To drive the coupling system into the strong coupling regime, the condition $\Omega >\bar{\gamma }=\left(\gamma_{p}+\gamma_{ex}\right)/2$ should be met [[28], [42]], where $\gamma_{p}$ and $\gamma_{ex}$ are the exciton linewidths of the bare bowtie structure and the free QD, respectively. This is a stricter criterion for strong coupling, with the establishment of Rabi oscillations in the time domain [28]. The lower dissipation of the plasmonic mode in the hybrid cavity contributes to achieving a larger Rabi splitting. Based on the above strong coupling criteria and the values extracted from the spectra, we find that 56% of the bowtie nanocavity coupling devices achieve strong coupling, while all hybrid nanocavity devices do. This fully demonstrates the effectiveness of our designed hybrid nanocavity in achieving strong coupling with single excitons in a single quantum dot.

### 3.3 Polarization-resolved measurements of coupled system

**Fig. 5:**
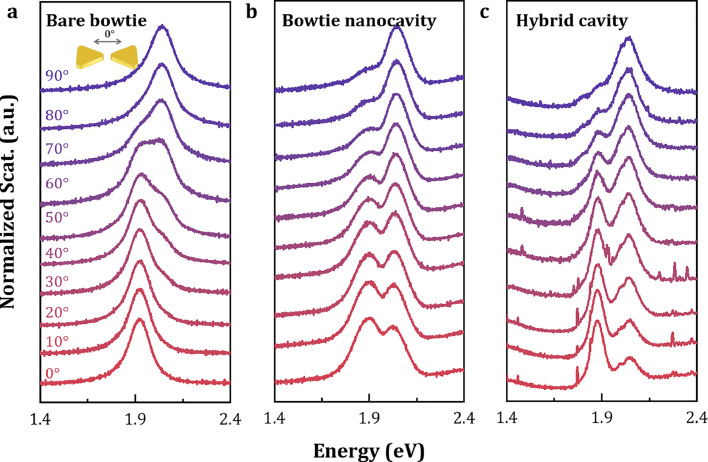
**Polarization-resolved scattering spectra.** (a) Polarization-dependent scattering spectra of the bare bowtie structure. (b-c) Polarization-dependent scattering spectra of (b) bowtie nanocavity and (c) hybrid cavity with a single quantum dot embedded. An obviously splitted feature is obtained when the polarization of incident light is aligned parallel to the long axis of the bowtie structure.

To verify that the observed spectral splitting originates from the interaction between the longitudinal plasmon mode of the bowtie structure and the QD, we performed polarization-dependent experiments. Here, the polarization of the excitation source was adjusted. Fig. 5a presents the polarization-dependent scattering spectra for a bare bowtie nanostructure. As the excitation polarization angle varies from $0^{\circ }$ to $90^{\circ }$, the plasmon mode transitions from the horizontal mode (1.92 eV) to the vertical mode (2.04 eV).

The polarization-dependent scattering spectra for a bowtie nanocavity and a hybrid nanocavity embedded with a single QD are shown in Fig. 5b and Fig. 5c, respectively. When the excitation polarization is parallel to the long axis of the bowtie structure (i.e., horizontal polarization), the spectra exhibit two distinct peaks, a consequence of the energy level splitting induced by strong coupling with gap mode of bowtie. As the polarization rotates vertically to the long axis (i.e. vertical polarization), the double-peak spectrum is gradually replaced by a single peak from the vertical mode. These results unequivocally demonstrate that the observed strong coupling phenomenon is attributed to the gap plasmon-SQD coupling, confirming the successful deposition of QDs within the nanocavities.

### 3.4 Single quantum dot emission and blinking

**Fig. 6:**
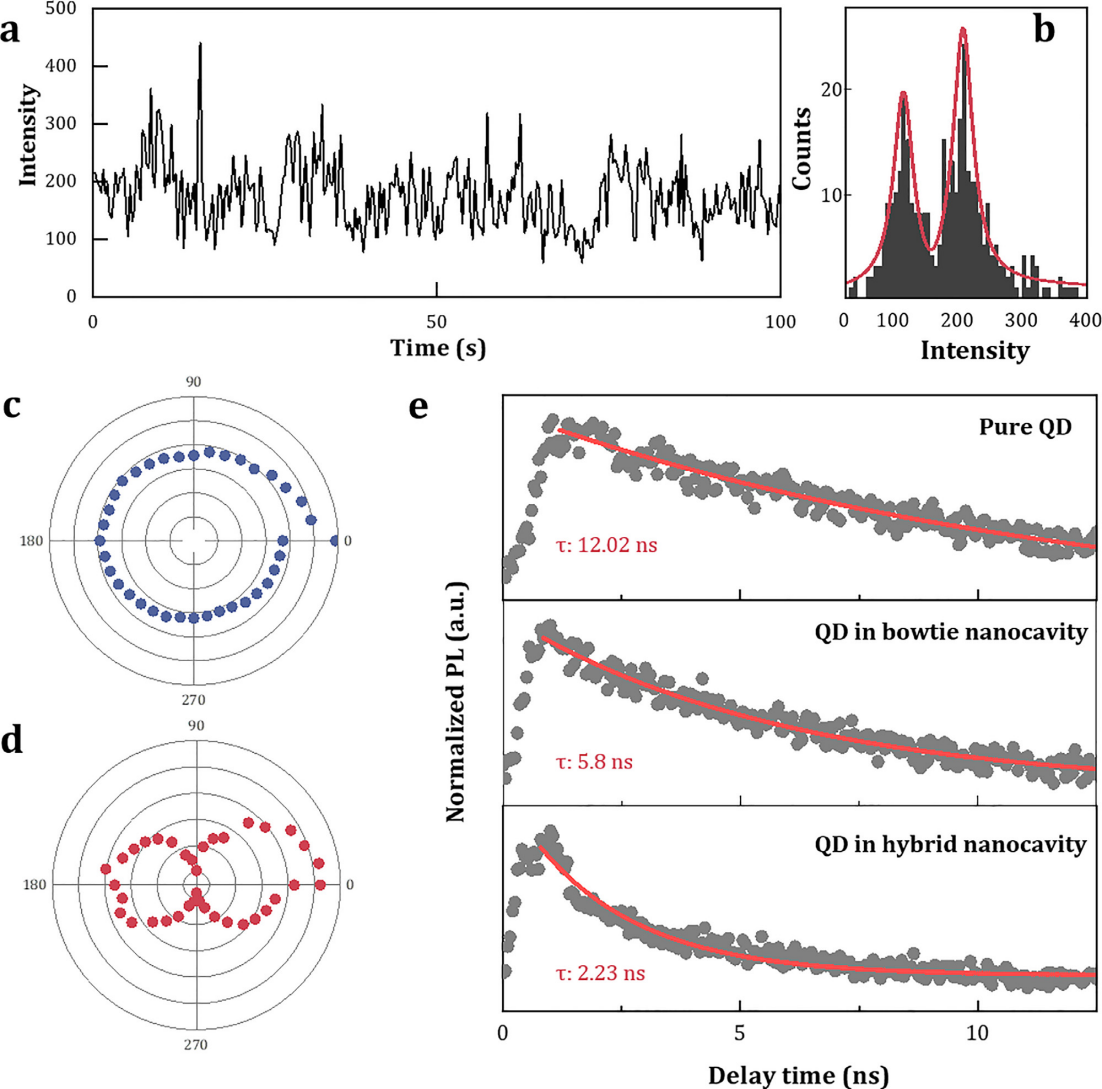
**Luminescence characteristics and dynamics of a single quantum dot.** (a) Time trace of the luminescence intensity from a single quantum dot. (b) The histogram of the luminescence intensity distribution exhibits a double-peak feature, confirming a single quantum dot. (c-d) Polarization-dependent luminescence spectra of free quantum dots and quantum dots within the cavity, with the latter displaying significant polarization anisotropy. (e) Time-resolved luminescence decay curves of a single quantum dot under different conditions.

The single-exciton emission efficiency of colloidal quantum dot (cQD) typically exhibits temporal fluctuations. This phenomenon, known as ”blinking,” serves as strong evidence for the existence of a single quantum system [[43], [44], [45]]. To investigate this phenomenon, we excited a single QD with a 532 nm continuous-wave (CW) laser and recorded the time trajectory of its PL intensity (Fig. 6a). The states corresponding to the intensities of 100 and 200 correspond to the “on” and “off” states of the QD blinking, providing strong evidence that only one QD is active within this hybrid system (Fig. 6b). The luminescence behavior obtained at other intensities could be due to spatial stray light.

For the coupling region between the bowtie structure and the QD, the fluorescence intensity with horizontal polarization is significantly higher than that with vertical polarization.Therefore, the polarization-dependent fluorescence spectrum can be used to verify whether the QD is located inside the cavity and coupled to the plasmons. We conducted a comparative studied the fluorescence properties of free QD and those located within the nanocavity, extracting the fluorescence peak intensities at various polarization angles, as shown in Fig. 6c and d. The fluorescence spectrum of single QD showing no significant polarization dependence (Fig. 6c). In contrast, the QDs within the nanocavity exhibit pronounced linear polarization. The fluorescence intensity reaches its maximum when polarized along the horizontal direction of the bowtie long aixs and its minimum when polarized vertically (Fig. 6d). This polarization anisotropy directly confirms the successful integration and efficient coupling of the QD with the nanocavity.

To further investigate the influence of plasmon modes on the excited-state dynamics of a single QD, we also conducted time-resolved fluorescence measurement. The typical fluorescence decay curves of a single QD in different conditions are displayed in Fig. 6e. All measurements were performed using the same excitation parameters. For isolated QDs on a glass substrate, the fluorescence decay curve exhibited an exponential decay behavior, with an average lifetime of approximately 12.02 ns. After confirming the presence of only a single QD within the nanocavity, we comparatively examined the modulation effect of the nanocavity condition on the QD’s emission dynamics. The results indicated that the fluorescence lifetimes of QDs in the bowtie nanocavity and the hybrid nanocavity were reduced to 5.8 and 2.23 ns, respectively.

When QD is present within the nanocavity, the increased local density of optical states (LDOS) in the nanocavity region leads to a reduced lifetime. This reduction is attributed to the contributions from both enhanced radiative emission (Purcell effect) and non-radiative energy transfer to the metal (quenching effect). A stronger electric field will further shorten the QD’s lifetime [46]. Therefore, the stronger electric field within the hybrid cavity is responsible for this observation, which is also consistent with the larger Rabi splitting observed in our scattering spectra.

## 4 Conclusion

We demonstrate an experimentally feasible scheme to achieve a robust strong coupling between a SQD and plasmonic nanocavity intermediated by a one-dimensional photonic crystal cavity. A Rabi splitting exceeding 170 meV is observed using dark-field scattering spectra. We further demonstrate that the stronger localized electric field within the hybrid cavity not only enhances the coupling strength but also, due to the more uniform field distribution, reduces the sensitivity of the coupling strength to the QD’s position within the cavity, thereby improving the device’s coupling strength uniformity. The robustness of such a strongly coupled system will improve room-temperature quantum devices based on a single emitter for potential applications.

## CRediT authorship contribution statement

**Bowen Fu:** Data curation, Writing – original draft, Writing – review & editing, Formal analysis. **Longlong Yang:** Data curation, Writing – review & editing. **Yu Yuan:** Data curation, Writing – review & editing. **Jingnan Yang:** Data curation, Writing – review & editing. **Hancong Li:** Data curation, Formal analysis, Writing – review & editing. **Zetao Fan:** Data curation, Writing – review & editing. **Sai Yan:** Data curation, Writing – review & editing. **Guowei Lu:** Data curation, Methodology. **Douguo Zhang:** Conceptualization, Resources. **Qihuang Gong:** Conceptualization, Resources, Writing – review & editing. **Xiulai Xu:** Conceptualization, Formal analysis, Funding acquisition, Writing – original draft, Writing – review & editing.

## Declaration of competing interest

The authors declare that they have no conflicts of interest in this work.
